# Characterization and Differential Cytotoxicity of Gramicidin Nanoparticles Combined with Cationic Polymer or Lipid Bilayer

**DOI:** 10.3390/pharmaceutics14102053

**Published:** 2022-09-27

**Authors:** Yunys Pérez-Betancourt, Rachel Zaia, Marina Franchi Evangelista, Rodrigo Tadeu Ribeiro, Bruno Murillo Roncoleta, Beatriz Ideriha Mathiazzi, Ana Maria Carmona-Ribeiro

**Affiliations:** Biocolloids Laboratory, Departamento de Bioquímica, Instituto de Química, Universidade de São Paulo, São Paulo 05508-000, Brazil

**Keywords:** antimicrobial peptide, antimicrobial cationic polymer, antimicrobial cationic lipid, nanoparticles, supported cationic bilayer on silica with or without gramicidin, scanning electron microscopy, dynamic light scattering, broad spectrum of antimicrobial activity, differential Gr nanoparticles/PDDA cytotoxicity

## Abstract

Gramicidin (Gr) nanoparticles (NPs) and poly (diallyl dimethyl ammonium) chloride (PDDA) water dispersions were characterized and evaluated against Gram-positive and Gram-negative bacteria and fungus. Dynamic light scattering for sizing, zeta potential analysis, polydispersity, and colloidal stability over time characterized Gr NPs/PDDA dispersions, and plating and colony-forming units counting determined their microbicidal activity. Cell viabilities of *Staphylococcus aureus*, *Pseudomonas aeruginosa*, and *Candida albicans* in the presence of the combinations were reduced by 6, 7, and 7 logs, respectively, at 10 μM Gr/10 μg·mL^−1^ PDDA, 0.5 μM Gr/0. 5μg·mL^−1^ PDDA, and 0.5 μM Gr/0.5 μg·mL^−1^ PDDA, respectively. In comparison to individual Gr doses, the combinations reduced doses by half (*S. aureus*) and a quarter (*C. albicans*); in comparison to individual PDDA doses, the combinations reduced doses by 6 times (*P. aeruginosa*) and 10 times (*C. albicans*). Gr in supported or free cationic lipid bilayers reduced Gr activity against *S. aureus* due to reduced Gr access to the pathogen. Facile Gr NPs/PDDA disassembly favored access of each agent to the pathogen: PDDA suctioned the pathogen cell wall facilitating Gr insertion in the pathogen cell membrane. Gr NPs/PDDA differential cytotoxicity suggested the possibility of novel systemic uses for the combination.

## 1. Introduction

Self-assembly is ubiquitous in nature and has been finding a myriad of strategic biomimetic applications in drug and vaccine delivery, some of them tailored as antimicrobial nanostructures able to overcome pathogen resistance to antibiotics [1,2]. Supramolecular nanostructures spontaneously assemble based on weak—relative to kT—interactions between the assembling molecules [2]. A remarkable example of resistance to cationic antimicrobials evolved in *Staphylococcus* sp. as a sensor system for cationic antimicrobial peptides (AMPs) consisting of a short and negatively charged extracellular loop of amino acid residues; the interaction with the cationic AMP would trigger the d-alanylation of teichoic acids and the lysylation of phosphatidylglycerol, resulting in a decreased negative charge of the bacteria and reduced affinity for cationic antimicrobials [3]. Besides the cationic antimicrobial peptides, many cationic nanostructures based on lipids or cationic antimicrobial polymers would also be sensed by this system due to their multipoint attachment to the negatively charged loop. As a possible alternative, neutral antimicrobial peptides and their self-assembled nanostructures may represent a suitable alternative to cationic antimicrobials. An important neutral antibiotic active against Gram–positive bacteria is gramicidin D [4,5]; its mechanism of action lies in destroying the ionic balance of the bacteria [6].

Gramicidin D (Gr) is produced as a mixture of six peptides where gramicidins A, B, and C represent 80, 4, and 16 % of the total composition, respectively [7]. The primary structure of gramicidin A has 15 amino acid residues, which include as the 11th residue a tryptophan; in gramicidin B, there is a phenylalanine, whereas, in gramicidin C, one finds a tyrosine. The tertiary structure of gramicidin A was determined at high resolution [8]. Gr selects cations for transport through its ion channel, and its dimers span across lipid membranes and adopt a β-helical conformation. These channels have been extensively studied not only as model ion channels [9] but also as a starting point for peptide synthetic chemistry in order to improve the chemical design for optimal activity and solubility at low toxicity against mammalian cells [10]. Artificial gramicidins have been designed aiming at a better understanding of the translocation mechanism of water molecules or ions through channels within lipid bilayers [11].

All gramicidins form channels with similar properties [12]. Gramicidins may contain three or four tryptophans per monomer, which in the channel form of gramicidin are positioned at the membrane/water interface [13,14]. Molecular dynamics simulations for Gr have shown the anisotropy of the electrostatic potential on the molecule: regions of negative electrostatic potential occurred in the channel and at the channel mouth electrostatically, driving the transport of cations through the channel [15]. The molecular shape of Gr beta-helix is conical so that the channel entrance corresponds to the inverted cone’s base (where the tryptophans are) facing the water phase [16]. Gr channel function is directly related to membrane thickness and the negative charge density at the channel entrance [17].

Bilayers of the antimicrobial and cationic lipid dioctadecyldimethylammonium bromide (DODAB) have successfully reconstituted functional Gr dimeric channels [6,18,19]. Aiming at strategic antimicrobial combinations of Gr and DODAB, our research was driven by two established pieces of evidence: (1) Gr exhibits high activity against Gram-positive bacteria and fungus but also high toxicity against mammalian cells [10,19,20]; and (2) DODAB bilayers display moderate activity against Gram-negative bacteria but poor activity against Gram-positive ones and fungus plus low toxicity against mammalian cells. Eventually, combining DODAB bilayer with Gr would produce a broadened spectrum of antimicrobial activity [19]. Several formulations based on DODAB bilayers have been developed in our laboratory for drug and vaccine delivery, such as the large DODAB vesicles, the DODAB bilayer fragments, the supported DODAB bilayers on polystyrene sulfate nanoparticles, or the supported DODAB bilayers on silica [21]. Here, we incorporate Gr in supported DODAB bilayers on silica to establish how DODAB would affect Gr antimicrobial activity against *S. aureus*; Gr incorporated in DODAB bilayers displayed lower activity against *S. aureus* in comparison with the Gr control (see the Results section in this work). This suggested that the strong interaction between Gr hydrophobic molecules and DODAB bilayers would prevent, to a certain extent, the Gr interaction with the bacteria cell membrane, leading to the need for promoting weaker interactions between Gr and its carrier.

As another possible active carrier for Gr, the cationic antimicrobial polymer poly (diallyl dimethyl ammonium) chloride (PDDA) has the advantage of being effective against Gram-negative bacteria and fungus [22]. Furthermore, PDDA displays affinity for proteins such as albumin [23,24] and ovalbumin [25], yielding hybrid PDDA/protein nanoparticles and revealing its affinity also for peptides due to weak non-covalent intermolecular interactions between the polymer and the peptide. In fact, combinations of polyelectrolytes and antimicrobial peptides have been reported as potent antimicrobial coatings on solid surfaces [26,27,28]. Lately, the literature has presented several effective combinations of antimicrobial agents, including membrane-disrupting cationic polymers and antibiotics against opportunistic bacteria [29], guanidinium and quaternary ammonium polymers acting synergistically by the translocation/precipitation of cytosolic components or disruption of the microbe membrane, respectively [30], and, antibiotics combined with biocompatible 2, 6-diamino chitosan that decreased by 2.4 logs the infective pathogens in vivo [31].

In this study, aiming at a broad and complete loss of viability of representative pathogens such as *P. aeruginosa*, *S. aureus*, and *C. albicans*, we prepared, characterized, and evaluated the antimicrobial activity of combinations of Gr/PDDA in water dispersions. Several antimicrobial peptides (AMP) self-assemble in aqueous solutions to yield supramolecular nanostructures [1,2,32], such as the fibrils resulting from Aβ peptide aggregation in Alzheimer’s disease hypothesized as a defense against microbes in vivo [32]. Here, we show that Gr molecules self-assemble in water yielding nanoparticles (NPs) that could be characterized regarding their physical properties by dynamic light scattering (DLS) and scanning electron microscopy (SEM); Gr NPs were spherical, displayed a negative zeta potential, and showed high colloid stability in water. Gr NPs showed high activity against *S. aureus* and *C. albicans* and low activity against Gram-negative bacteria. Taking advantage of the high PDDA activity against Gram-negative bacteria [22,33], adding PDDA to Gr NPs caused the complete loss of *P. aeruginosa* viability and reduced the Gr and PDDA doses required for complete microbicidal effect. The low cytotoxicity against mammalian cells and the optimal activity of Gr NPs and Gr NPs/PDDA against the microbia tested indicated the potential of such combinations for systemic uses deserving further evaluation in vivo.

## 2. Materials and Methods

### 2.1. Materials

D-glucose, poly (diallyl dimethylammonium chloride) (PDDA) 35% *w*/*v* with very low molecular weight (<100,000) was obtained from Sigma (Steinheim, Germany), gramicidin D (a peptide mixture consisting mostly of Gr A), ethanol, chloroform, 2,2,2-trifluoroethanol (TFE), and Mueller–Hinton agar (MHA) were purchased from Sigma-Aldrich (St Louis, MO, USA). KCl and cationic lipid dioctadecyldimethylammonium bromide (DODAB) were from Sigma (St. Louis, MO, USA); SiO_2_, trade name AEROSIL OX-50, was supplied by Degussa (Frankfurt, Germany) and showed nanoparticles with 50 nm of mean diameter by transmission electron microscopy (TEM); the silica dispersion yielded 26.00 m^2^/g of specific surface area (BET technique). SiO_2_ was dispersed in doubly distilled, deionized water or in a water solution of KCl (1 mM).

### 2.2. Preparation of Gramicidin and Gramicidin/Poly (Diallyldimethylammonium Chloride) Dispersions in Water

Gramicidin (Gr) and PDDA stock solutions at 6.4 mM Gr and 10 mg/mL PDDA, respectively, were used to prepare 2 mL of dispersions in ultrapure water to yield the desired final concentrations. The final TFE concentration was kept at 1% of the final volume by adding 0.02 mL of appropriate Gr solutions in TFE to 2 mL pure water under stirring by vortexing for 30 s.

Gr/PDDA dispersions were prepared by adding 0.05 mL of a stock PDDA solution in water (10 mg/mL) to the previously prepared Gr dispersion (2 mL) also under stirring by vortexing for 30 s.

### 2.3. Preparation of Supported Dioctadecyldimethylammonium Bromide (DODAB) Bilayers on Silica Particles with or without Gramicidin Inserted in the DODAB Bilayer

DODAB lipid films on the bottom of assay tubes were obtained from vaporization of chloroform DODAB solutions [21] before adding silica 2 mg/mL, ultrasonically dispersing silica nanoparticles in the presence of the DODAB films, and centrifuging at 10,000× *g* in a microcentrifuge for collecting the supernatant. The tube was heated at 56 °C for 1 h for obtaining the supported DODAB bilayers on silica. An aliquot of the gramicidin stock solution was then added, and the tube heated for another hour. DODAB, Gr, and silica concentrations in the assay tube were 0.5 mM, 0.05 mM, and 2 mg/mL, respectively. Under these experimental conditions, all silica nanoparticles were covered by a DODAB bilayer able to provide adequate microenvironment for Gr [21].

SiO_2_/DODAB/Gr nanoparticles had their physical properties and antimicrobial activity determined as described below.

### 2.4. Determination of Physical Properties of Gr, Gr/PDDA, or SiO_2_/DODAB/Gr Dispersions

Physical characteristics of nanoparticles were determined by dynamic light scattering (DLS) such as the zeta-average diameter (Dz), the zeta potential (ζ), the polydispersity (P), and the conductance (G) using a Brookhaven apparatus (Zeta-Plus Zeta Potential Analyzer, Brookhaven Instruments Corporation, Holtsville, NY, USA) equipped with a 677 nm laser. The principles of DLS were explained in detail beforehand [34]. Briefly, the relationship between Dz and the particle diffusion coefficient (D) is the Stokes–Einstein equation, Dz = kT/(3πηD), where k is the Boltzmann’s constant, T is temperature in Kelvin, and η is the viscosity of the medium. The equipment software algorithm was the non-negatively constrained least squares (NNLS) for multimodal distributions [35]. Size distributions allowed us to obtain polydispersities (P) related to the width of the size distribution. The zeta potential (ζ) was determined from the Smoluchowski equation, ζ = μη/ε, where electrophoretic mobility is µ in 1 mM NaCl, η is the medium viscosity, and ε the medium dielectric constant.

### 2.5. Determination of Colloidal Stability

The colloidal stability of Gr or Gr/PDDA dispersions was evaluated at 0, 1, and 24 h after preparation by DLS and turbidimetry at 400 nm. Data were presented as zeta-average diameter (Dz), zeta potential (ζ), polydispersity (P), and absorbance at 400 nm over time.

### 2.6. Determination of Circular Dichroism Spectra for Gramicidin in Ethanol, Trifluoroethanol, Water, and Poly (Diallyldimethylammonium) Chloride Solutions

Circular dichroism spectroscopy was performed at 25 °C by means of a 720 Spectropolarimeter (Jasco Inc, Tokyo, Japan) in a 0.1 cm quartz cuvette with the following parameters: 0.5 nm wavelength increments; 4 s response; wavelength range of 200–280 nm; 100 nm/min scan rate; averaging 5 scans per spectrum; 10 m deg sensitivity. Background corrections were obtained subtracting blanks in the absence of Gr such as PDDA solution or Gr solvent. Spectral shape was preserved by smoothing. Ellipticities θ (deg dmol^−1^ cm^2^) were a function of wavelength.

### 2.7. Scanning Electron Microscopy for Gr or Gr/PDDA Dispersions

Dispersions were prepared at 0.05 mM Gr or 0.05 mM Gr/0.05 mg/mL PDDA in water and further diluted in water by a factor of 1:2 before placing them on coverslips for drying overnight at room temperature. Thereafter, samples were coated with a gold layer using a Leica EM SCD 050 sputtering apparatus and examined using a Jeol JSM-6460LV scanning electron microscope for obtaining the micrographs.

### 2.8. Determination of Cell Viability for Staphylococcus aureus, Candida albicans, and Pseudomonas aeruginosa over a Range of Gramicidin, Gramicidin/Poly (Diallyldimethylammonium) Chloride, or Dioctadecyldimethylammonium Bromide Concentrations

Microbial strains from American Type Culture Collection (ATCC) were *Staphylococcus aureus* ATCC29213 or *Candida albicans* (ATCC 90028). They were grown from stocks at −20 °C in appropriate solutions. Reactivation for each strain took place separately by streaking the microbes on Mueller–Hinton agar (MHA) plates for 18–24 h/37 °C incubation. Thereafter, some colonies were added to 0.264 M D-glucose isotonic solution and the turbidity at 625 nm of suspensions was adjusted to 0.5 of the McFarland scale. The 0.264 M D-glucose solution was used due to inactivation of cationic antimicrobials by ionic strength or other negatively charged components of the culture medium (e.g., amino acids or polysaccharides). Viability curves were obtained with 0.1 mL of the cell suspensions containing 10^7^–10^8^ colony-forming units per mL (CFU·mL^−1^) mixed with 0.9 mL of antimicrobial dispersions diluted in the same D-glucose solution and allowed to interact for 1 h. Next, 0.1 mL was either withdrawn from the mixtures for plating or further diluted before plating. The medium used for plating was MHA. Plates were then incubated (37 °C/24 h) before counting (CFU/mL) and plotting the results on a logarithmic scale vs. the concentration of the agent under test. No counting was considered as 1 so that log CFU·mL^−1^ could be taken as zero.

*Pseudomonas aeruginosa* PA14, full name UCBPP-PA14 [36,37], was a kind gift from Dr. Regina Lúcia Baldini from our department and was kept frozen in Luria–Bertani (LB) medium containing 20% glycerol. Bacteria reactivation and testing proceeded as described above.

## 3. Results

### 3.1. Assembly of Gramicidin and Gramicidin/Poly (Diallyldimethylammonium) Chloride in Water as Nanoparticles

**Figure 1:**
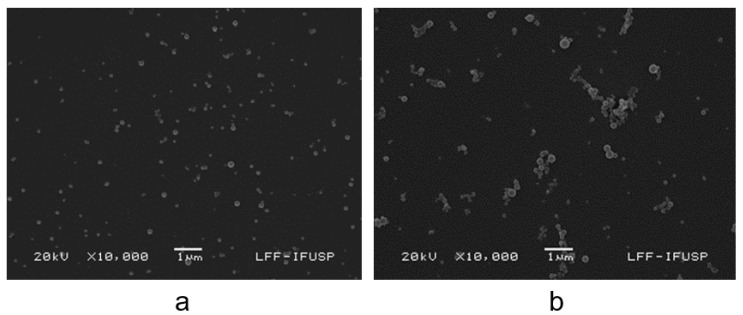
Scanning electron micrographs of gramicidin (Gr) dispersions at 0.05 mM Gr in pure water (**a**) or in 0.05 mg/mL poly (diallyldimethyl ammonium chloride) (PDDA) solution (**b**).

The SEM micrographs for Gr dispersions in water showed the occurrence of spherical Gr NPs (Figure 1a). For Gr/PDDA dispersions in pure water, similar spherical Gr NPs displayed an apparent aggregation (Figure 1b). This apparent aggregation of Gr NPs seen in the Gr/PDDA dispersion on Figure 1b was not confirmed by dynamic light scattering (DLS) and might have derived from drying the Gr/PDDA dispersion before depositing the gold coating for SEM visualization. The mean hydrodynamic diameter (Dz) for 0.05 mM Gr/0.05 mg/mL PDDA NPs was 240 nm.

Table 1 shows the macroscopic features and physical properties of Gr, PDDA, and Gr/PDDA dispersions in pure water. Gr in water yielded turbid dispersions, meaning that these dispersions were made of Gr aggregates shaped as nanostructures able to scatter the incident light. DLS confirmed the particulate nature of these Gr dispersions in water; at 0.1 mM Gr, Gr nanoparticles (NPs) with 159 ± 1 nm of the mean hydrodynamic diameter displayed a negative zeta potential of −26 ± 3 mV (Table 1). Gr intermolecular aggregation in water was possibly driven by the hydrophobic effect due to nonpolar moieties in the amino acid sequence of Gr, such as those of isoleucine and valine. The negative zeta potential of Gr NPs was in agreement with studies by Chen and Wei using molecular dynamics simulations to predict regions of negative electrostatic potentials at the Gr channel and channel mouth [15]. The negative zeta potential determined at the shear plane of the Gr NPs might be related to the Gr anisotropy of electrostatic potential distribution on the Gr molecule, corroborating the molecular dynamics simulation of Gr reported by Chen and Wei [15]. Furthermore, the conical geometry of the Gr molecule with tryptophans at the cone’s base [16] might have favored aggregation as nanoparticles with tryptophans at the nanoparticle/water interface. Inverted Gr cones could indeed aggregate to yield nanoparticles. Gr NPs in water could only be obtained from Gr stock solutions in TFE, where Gr assumes the beta-helix conformation. From stock solutions of Gr in ethanol, where Gr molecules are intertwined, fibers and filaments could be visualized given enough time after addition of the Gr ethanol solution to water (not shown). The solvent-dependent self-assembly of gramicidin A has been previously described [38]. Gr added to solvents with increasing polarity, such as ethanol, TFE, and water, would undergo oligomerization with the highest frequency of oligomers occurring in the more polar solvent. Thus, the formation of Gr NPs in pure water is consistent with water’s high dielectric constant and polarity so that water would be a poor Gr solvent promoting Gr oligomerization.

Adding PDDA (0.25 mg/mL) to Gr nanoparticles (Gr NPs) (0.1 mM Gr) changed the zeta potential from negative to positive values and increased particle size and polydispersity (Table 1). At this relatively large PDDA concentration, one can expect bridging flocculation joining one or more Gr nanoparticles [39], thereby increasing the mean particle size in the dispersion. Table 1 also shows that PDDA hydrophilic cationic polymer is readily soluble in water and yields transparent water solutions.

**Figure 2:**
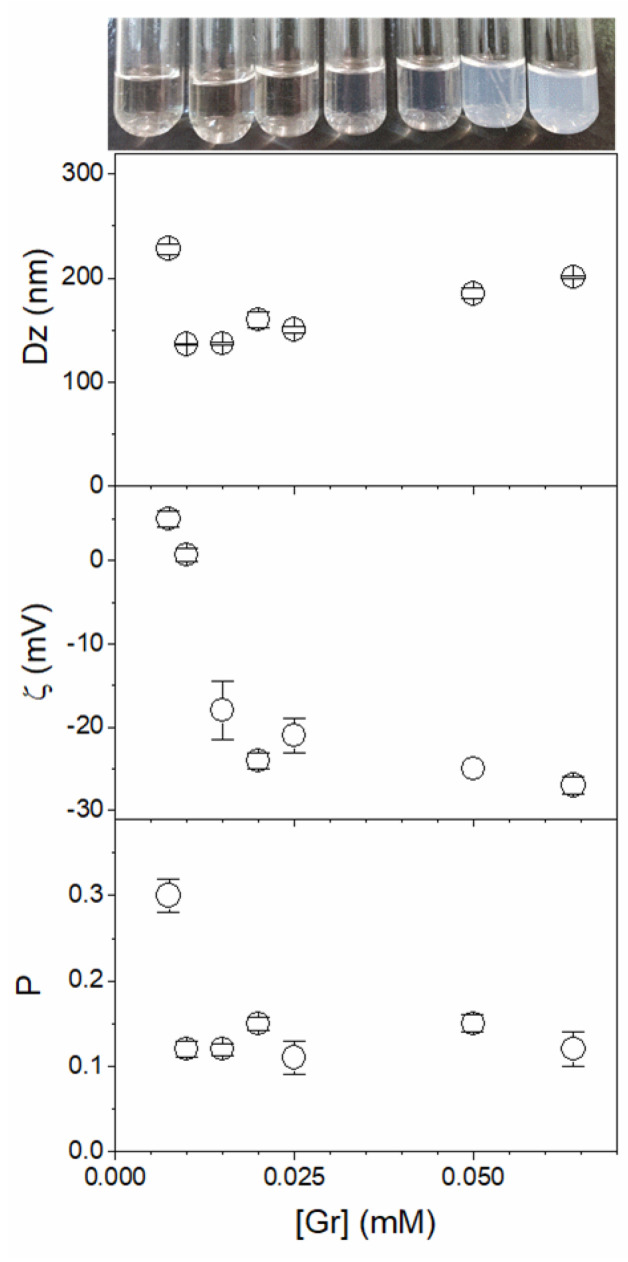
Effect of gramicidin (Gr) concentration on the physical properties of Gr dispersions in water and 1% trifluoroethanol. Measurements were performed 30 min after preparation of the Gr dispersions. Physical properties were the macroscopic aspect, the mean z-average diameter (Dz), the zeta potential (ζ), and the polydispersity (P). All measurements represent a mean value ± the mean standard deviation.

Figure 2 shows the effect of Gr concentration on the physical properties of Gr NPs. With exception of the first point at a very low Gr concentration (0.005 mM), Dz and zeta potential for the Gr NPs increased with Gr concentration. Therefore, increasing the number of Gr molecules in the nanoparticle might have increased not only their size but also the negative charges available for colloidal stabilization. This stability was favored not only by the negative charges but also by the nature of ethanolamine moieties that are covalently bound to the Gr molecule terminus. The hydroxyl moieties in ethanolamine may form hydrogen bonds with water and are possibly occupying the outer surface of the Gr nanoparticle. The origin of the negative zeta potential at the shear plane of the Gr NPs could be understood from molecular dynamics simulations of gramicidin A, with negative electrostatic potentials at the channel’s mouth and inside the channel as the driving forces for the transport of cations through the Gr channel [15]. This would also explain the negative zeta potential obtained for the Gr NPs (Table 1) despite the net charge equal to zero depicted by the primary chemical structure of Gr.

The polydispersity (P) of Gr NPs remained constant as a function of Gr concentration, revealing a good colloidal stability and an absence of aggregation between nanoparticles (Figure 2). Taking this result in combination with the increase in particle size (Dz) upon increasing Gr concentration reveals that the increase in Dz is due to the increase in the number of Gr molecules in each nanoparticle instead of interparticle aggregation.

**Figure 3:**
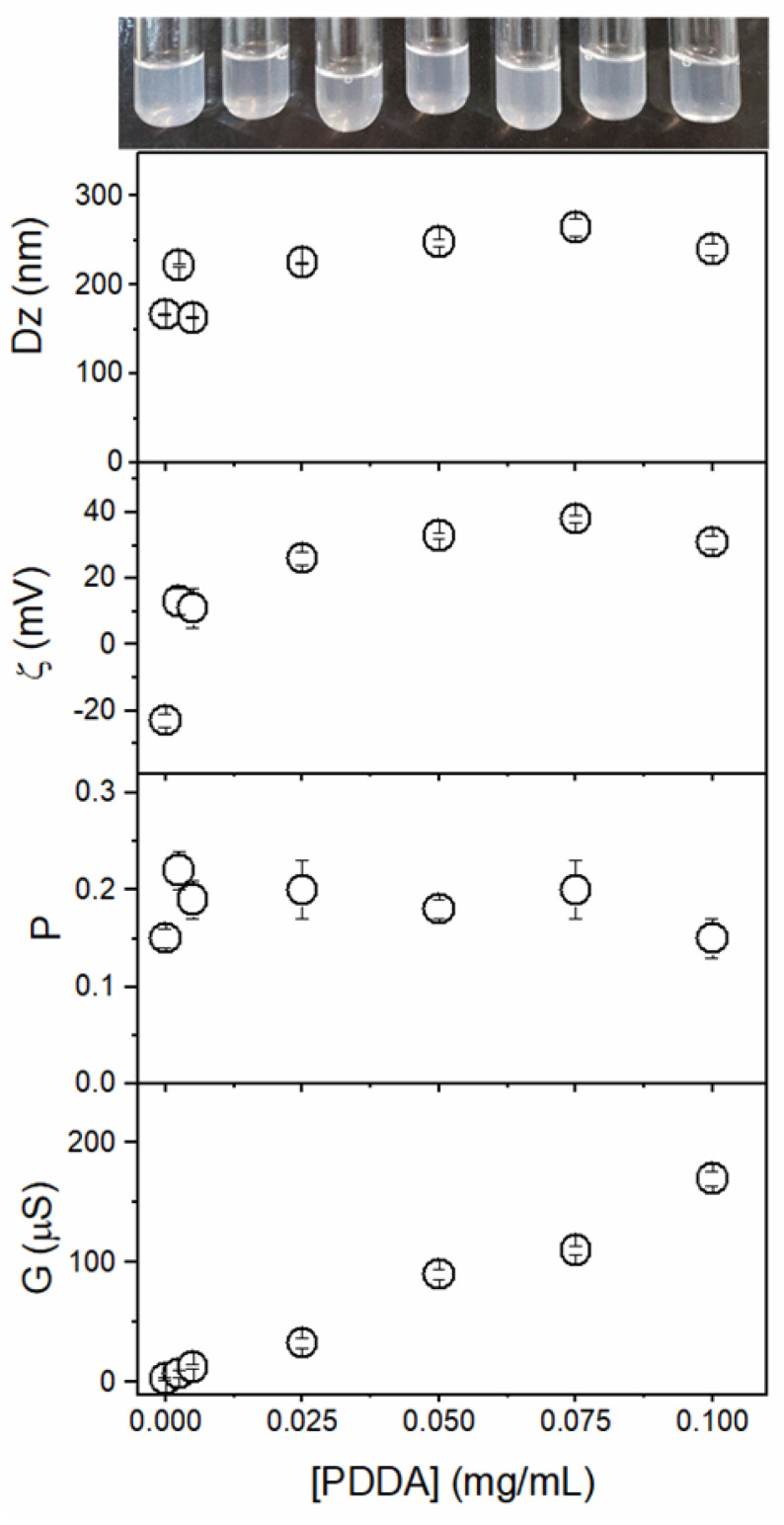
Effect of poly (diallyldimethyl ammonium chloride) (PDDA) concentration on the macroscopic aspect and physical properties of gramicidin (Gr)/PDDA dispersions at 0.05 mM Gr. Physical properties were the mean z-average diameter (Dz), the zeta potential (ζ), the polydispersity (P), and the conductance (G). All measurements represent a mean value ± the mean standard deviation.

Figure 3 shows the effect of [PDDA] (0–0.1 mg/mL PDDA) at 0.05 mM Gr on Dz, P, zeta potential, and the conductance of the Gr/PDDA dispersions. In addition, the photos evidence the turbid but homogeneous features of the Gr/PDDA dispersions in pure water. In absence of PDDA, the Gr NPs exhibit a slightly negative zeta potential that was easily changed to positive values with tiny amounts of PDDA. The mean zeta potential of Gr/PDDA in the dispersions was positive; conductance increased linearly with [PDDA] (Figure 3). This showed that Gr NPs did not hamper PDDA mobility in the Gr NPs/PDDA dispersions.

**Figure 4:**
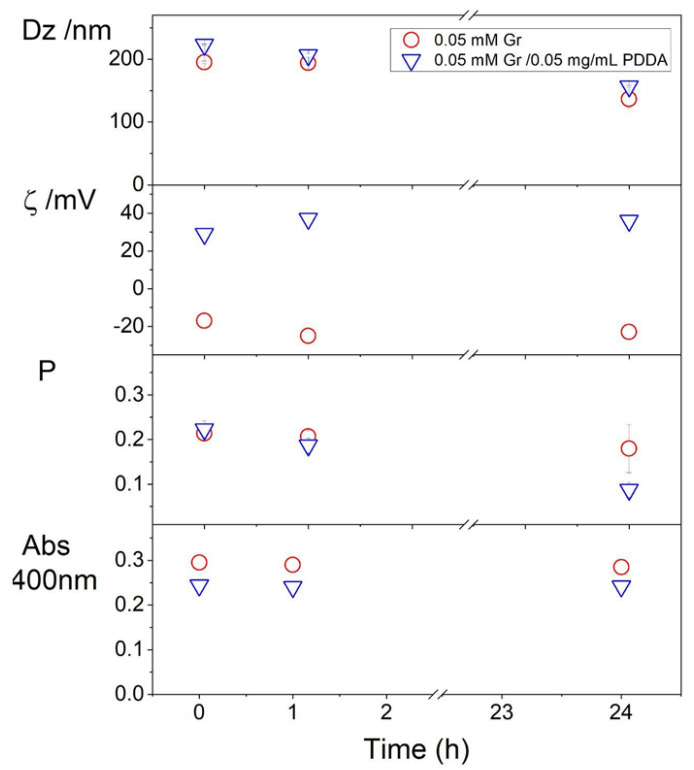
Colloidal stability of gramicidin (Gr) nanoparticles (NPs) in the absence or presence of poly (diallyldimethyl ammonium chloride) (PDDA). Mean z-average diameter (Dz), zeta potential (ζ), polydispersity (P), and turbidity at 400 nm (Abs 400 nm) of gramicidin (Gr) nanoparticles in water or in 0.05 mg/mL PDDA as a function of time.

The colloid stability of Gr/PDDA dispersions was assessed from dynamic light scattering and turbidity at 400 nm measurements as a function of time over 24 h. Physical properties remained basically unchanged revealing the good colloid stability of the dispersions (Figure 4).

**Figure 5:**
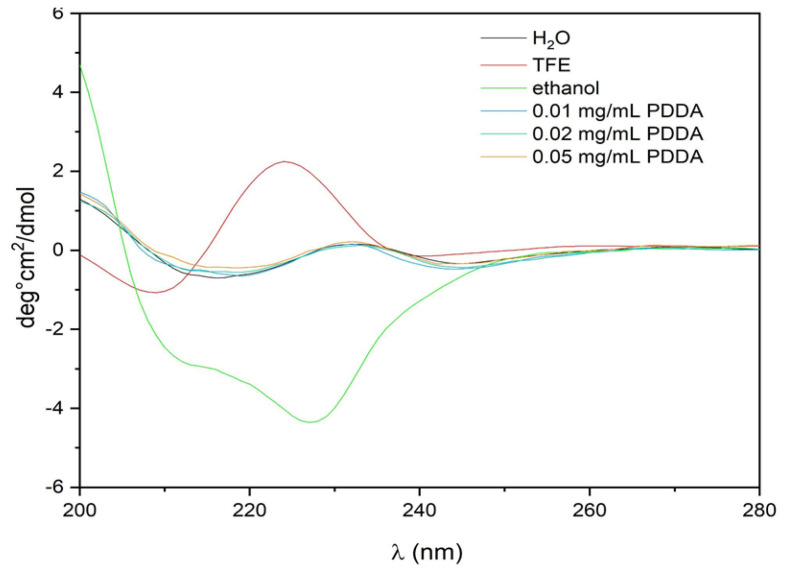
Circular dichroism spectra of 0.02 mM gramicidin (Gr) at 25 °C in different media. Aliquots (0.02 mL) of Gr stock solutions (6.4 mM) in trifluoroethanol (TFE) or ethanol were added to 2.0 mL of water, TFE, or ethanol. One should notice that in water or in aqueous PDDA solutions, Gr spherical NPs yielded the same spectrum. Alternatively, aliquots of poly (diallyldimethyl ammonium chloride) (PDDA) stock solution (10 mg/mL) were added to Gr dispersion under stirring by vortexing to yield 0.01, 0.02, and 0.05 mg/mL PDDA.

To gain some insight into the secondary structure or conformation of the Gr peptide in the Gr NPs/PDDA dispersions, the circular dichroism spectra of Gr NPs in the presence and absence of PDDA were obtained (Figure 5). The Gr peptide assumes an intertwined conformation in ethanol and a beta-helix turn in TFE. In the Gr NPs with or without PDDA, there was a significant reduction in intensity of the circular dichroism of the Gr molecules, possibly due to their tight packing in the Gr NPs. Gr NPs in the presence or absence of PDDA yielded the same reduced CD spectrum. PDDA did not contribute to the spectra of the Gr NPs; however, the type of assembly for Gr in the NPs did indeed reduce the CD signal and to a certain extent abolished the secondary structure of Gr.

**Figure 6:**
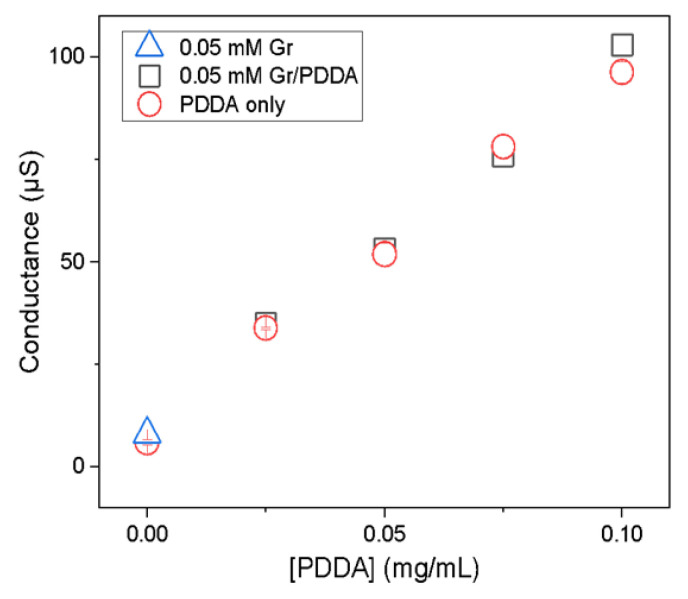
Conductance of a 0.05 mM gramicidin (Gr) nanoparticles (NPs) dispersion in the absence (∆) or presence of poly (diallyldimethyl ammonium chloride) (PDDA) (□). PDDA conductance in absence of Gr was also measured over a range of [PDDA] (o).

Figure 6 shows the conductance (G) of Gr NPs and Gr NPs/PDDA as a function of [PDDA] at 0.05 mM Gr in water. The dispersion of Gr NP yielded a conductance value close to that of pure water. The Gr/PDDA dispersions yielded conductance values identical to those of the corresponding PDDA solutions. The data for Gr NPs, PDDA, and Gr NPs/PDDA conductance show that PDDA and Gr NPs interact weakly. The complete immobilization of PDDA in polymeric NPs abolished PDDA conductance in the dispersion [34]. Conversely, a weak interaction of PDDA with the Gr NPs did not affect its conductance.

### 3.2. Incorporation of Gramicidin in Dioctadecyldimethylammonium Bromide Bilayers Reduces Gramicidin Antimicrobial Activity against Staphylococcus aureus

**Figure 7:**
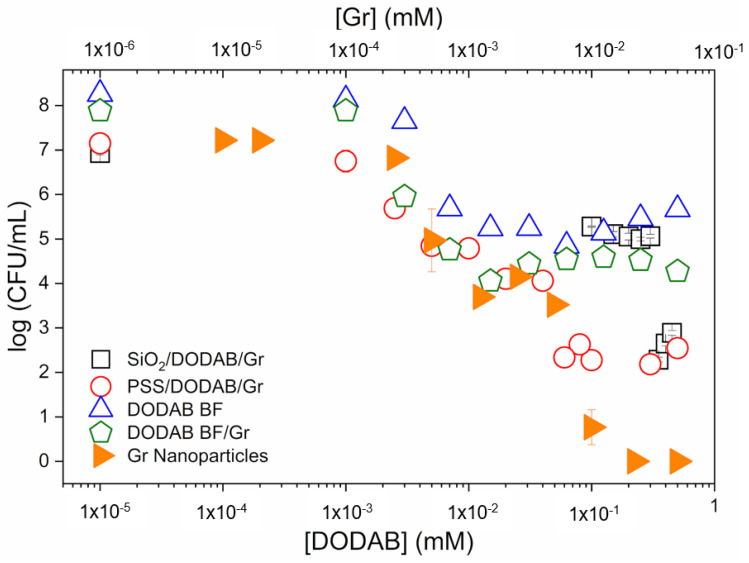
Cell viability of *Staphylococcus aureus* (10^7^–10^8^ CFU/mL) after interacting for 1 h with gramicidin (Gr) nanoparticles or other Gr formulations in dioctadecyldimethylammonium bromide (DODAB) bilayers. Cell viability in the presence of Gr in DODAB supported bilayers on silica (SiO_2_/DODAB/Gr) [21], Gr in DODAB supported bilayers on polystyrene sulfate (PSS) nanoparticles (PSS/DODAB/Gr) [40], and DODAB bilayer fragments (DODAB BF [41] or DODAB BF/Gr [19]), all of them incorporating Gr. In the DODAB BF dispersions, Gr dimers in the channel conformation have been previously described [19,40]. The SiO_2_/DODAB/Gr stock dispersion was prepared at 2 mg/mL silica, 0.5 mM DODAB, and 0.05 mM gramicidin, yielding Dz = 280 ± 5 nm, P = 0.20 ± 0.02, and ζ = 45 ± 4.

Figure 7 shows the relative efficacy of Gr formulations developed in our group against *S. aureus*. Different Gr formulations with bilayers of the cationic antimicrobial lipid DODAB, supported or non-supported by nanoparticles, were used to incorporate Gr and to determine antimicrobial activity against *S. aureus* (Figure 7). The cationic lipid bilayer of DODAB, similarly to the cationic polymer PDDA, bears quaternary ammonium moieties well known for their activity against Gram-negative bacteria but yielding a poor performance against Gram-positive ones such as *S. aureus*. Gr dimeric channels found a very appropriate microenvironment in the DODAB bilayer prepared as large DODAB vesicles or bilayer fragments [19,40]; a strong interaction with DODAB hampered to a certain extent the Gr interaction with the coccus [19,40]. With Gr insertion in supported DODAB bilayers on PSS NPs [40] or on silica [21], despite some improvement in the activity against *S. aureus* as compared to the free DODAB bilayers, the complete killing of the pathogen did not take place (Figure 7). The highest activity against *S. aureus* was the one achieved by the Gr NPs by themselves, reaching a 7-log reduction in cell viability (Figure 7). Therefore, the insertion of Gr in supported or non-supported DODAB bilayers diminished Gr activity against *S. aureus* as compared to the Gr NPs control.

### 3.3. Combinations of Gramicidin Nanoparticles (Gr NPs) and Poly (Diallyldimethyl Ammonium Chloride) (PDDA) in Water Dispersions Exhibit High and Broad Microbicidal Activity in Absence of Cytotoxicity against Mammalian Cells at Reduced Doses of Both Antimicrobials

**Figure 8:**
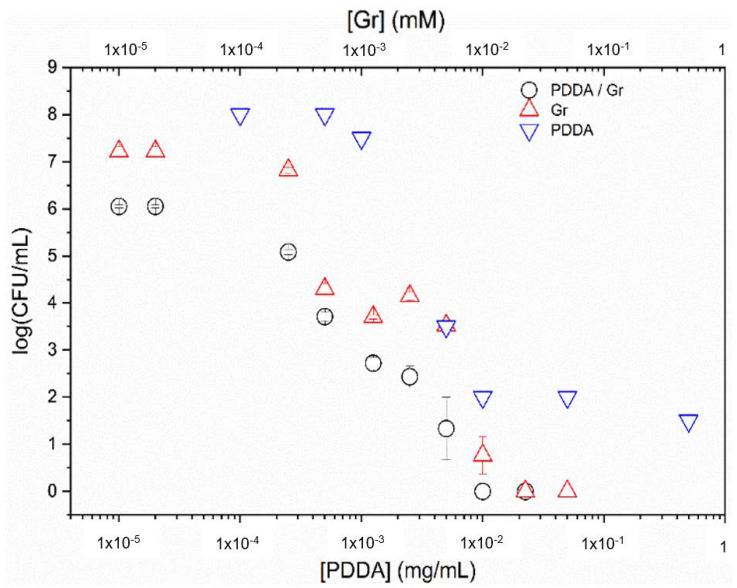
Cell viability of *Staphylococcus aureus* (10^6^–10^8^ CFU/mL) after interacting for 1 h with gramicidin nanoparticles (Gr NPs), poly (diallyldimethyl ammonium chloride) PDDA/Gr NPs, or PDDA solutions over a range of Gr and/or PDDA concentrations. Data for cell viability over a range of [PDDA] were taken from [22].

In Figure 8, the Gr NPs combined with PDDA were evaluated against *S. aureus.* The complete loss of cell viability for the combination occurred with the minimal microbicidal concentration (MMC) of 0.01 mM Gr/0.01 mg/mL PDDA (black hollow circles).

Gr NPs only (red triangles) showed MMC equal to double the MMC obtained for the Gr NPs/PDDA dispersion (black hollow circles).

PDDA only (inverted blue triangles) did not cause a complete loss of *S. aureus* viability, as reproduced from [22].

Against *S. aureus*, Gr NPs/PDDA dispersion yielded the lowest MMC values for Gr. This was half the dose for the Gr NPs: MMC = 0.02 mM (Figure 8). Given Gr toxicity, Gr dose reduction in the combination with PDDA might be a significant advantage.

**Figure 9:**
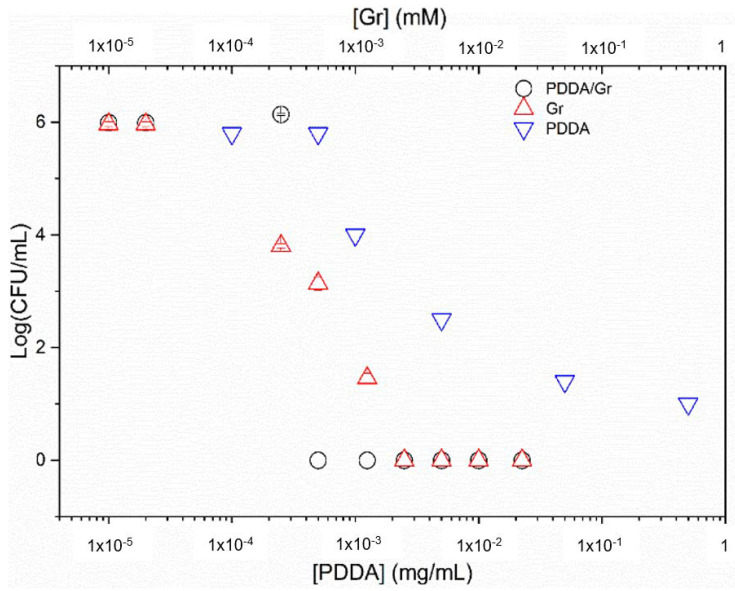
Cell viability of *C. albicans* (10^6^ CFU/mL) after interacting for 1 h with gramicidin (Gr) nanoparticles (NPs), poly (diallyl dimethyl ammonium chloride) (PDDA) solutions, or Gr NPs/PDDA dispersions over a range of Gr and/or PDDA concentrations. Data for cell viability in the presence of PDDA only, over a range of PDDA concentrations, were reproduced from [22].

In Figure 9, Gr NPs, PDDA, and Gr NPs/PDDA dispersions were tested against *Candida albicans*. There was a reduction in Gr and PDDA MMC required for the complete loss of fungus viability for the Gr NPs/PDDA combination, with 0.5 µg·mL^−1^ of each antimicrobial agent, comparing favorably with the required dose of 2.5 µg·mL^−1^ of Gr NPs to reduce fungus viability to zero or with PDDA only that was unable to completely kill at 500 µg·mL^−1^ (Figure 9). Testing covalently bound Gr in gold coatings yielded only a 90% reduction in fungus viability [42].

For Gr NPs, the complete loss of *C. albicans* viability in Figure 9 possibly reflected the high availability of Gr molecules to interact with the fungus from the Gr NPs. The combined action of Gr NPs and PDDA caused the lethal effect on the fungus at lower doses than those for Gr only, showing that the cationic polymer is possibly paving the way for the antimicrobial peptide to the fungus cell membrane across its rough cell wall. One should also notice the high dose reduction achieved

**Figure 10:**
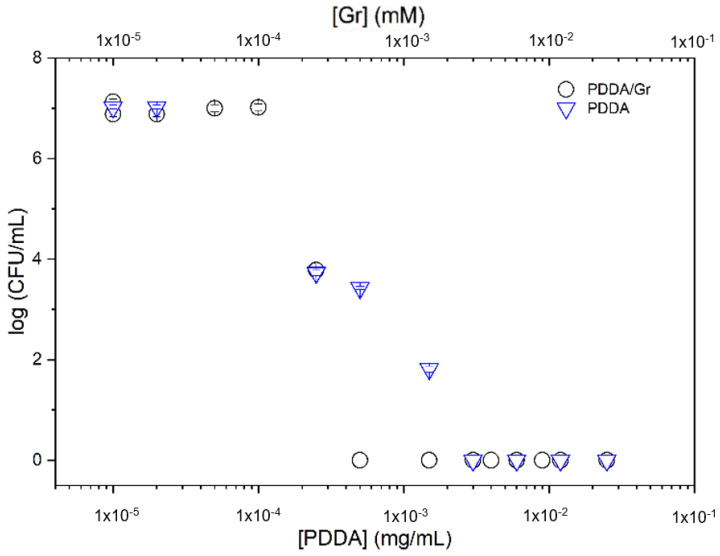
Cell viability of *Pseudomonas aeruginosa* PA14 (10^7^ CFU/mL) after interacting for 1 h with poly (diallyl dimethyl ammonium chloride) (PDDA) solutions (blue inverted triangles) or gramicidin nanoparticles Gr NPs/PDDA (black hollow circles) over a range of Gr and/or PDDA concentrations.

The cell viability of *Pseudomonas aeruginosa* PA14 [36,37], a Gram-negative bacterium, was determined after a 1h interaction between the bacteria and PDDA or Gr NPs/PDDA over a range of concentrations (Figure 10). Similarly to the results for *C. albicans*, the combination with 0.5 µg·mL^−1^ reduced the MMC of PDDA from 3 µg·mL^−1^ (PDDA only) to 0.5 µg·mL^−1^ PDDA (Gr NPs/PDDA). Against a multidrug resistant *P. aeruginosa* strain, the literature has reported 1.5 µg·mL^−1^ as the MMC for PDDA; against a multidrug resistant Gram-negative *Klebsiella pneumonia*, the MMC for PDDA was 0.9 µg·mL^−1^ [33].

The Gr NPs/PDDA combination implements the activity of both antimicrobial agents. Gr, poorly active against Gram-negative bacteria [10,19], further increases the already high PDDA activity against them (Figure 10).

PDDA against Gram-positive bacteria (Figure 8) and *C. albicans* (Figure 9) further increases the already high Gr activity against them. Gr NPs/PDDA can be pointed out as a valuable combination for reducing the doses of both antimicrobial agents and deserves further investigation on its efficacy and toxicity in vivo.

Table 2 summarizes the MMC data and activity for Gr NPs, PDDA, and Gr NPs/PDDA against mammalian and microbial cells. One should notice the importance of obtaining and expressing cell viabilities as log CFU/mL in order to gain insight into the real potency of each antimicrobial separately and in the combinations; expressing cell viability in percentiles may not give a complete picture of the magnitude of their activity.

Against *E. coli*, Gr NPs displayed a poor antimicrobial effect, decreasing viability by 0.3 logs (equivalent to 50% of cell viability) at 5 μM Gr [19]. This contrasted with PDDA activity against this same bacterial strain, reducing cell viability by 8 logs from 5 μg/mL PDDA [22].

The combination Gr NPs/PDDA at 0.5 μM Gr and 0.5 μg/mL PDDA reduced *P. aeruginosa* viability by 8 logs (Table 2). Thus, Gr significantly contributed to a reduction in the PDDA dose required for complete bacterial killing.

Concentrations that completely kill bacteria and fungus remained well below the concentrations that induced significant loss of mammalian cell viability (Table 2). The differential cytotoxicity shown by the antimicrobial agents may become a valuable asset for further applications against infectious diseases. The mechanism by which the peptide and the cationic polymer exert their harmonic and complementary activity might be related to the possibility of weak interactions between both agents allowing their facile disassembly in front of the pathogens, as discussed in the next section. After disassembly, PDDA suctions the biopolymers of the cell wall facilitating Gr access to the bacterial membrane, as discussed in the next section.

## 4. Discussion

The particulate nature of gramicidin D dispersions in pure water was revealed from size determinations, zeta potential evaluation, polydispersity, morphology from scanning electron microscopy, and colloid stability analysis over time by dynamic light scattering techniques. Given the high activity of Gr against Gram-positive bacteria, e.g., *S. aureus* (Figure 7) and low activity against Gram-negative ones, e.g., *E. coli* [19], here the combinations of Gr NPs with the cationic antimicrobial polymer PDDA were aimed at broadening the spectrum of Gr antimicrobial activity, since PDDA showed high activity against *E. coli* [22] and other Gram-negative bacteria [33].

The Gr NPs/PDDA combination effectively yielded the complete loss of cell viability against 10^6^–10^8^ cells/mL over a 1 h interaction time between the dispersions and microbe (*Pseudomonas aeruginosa*, *S. aureus*, and *C. albicans*) (Figure 8, Figure 9 and Figure 10, Table 2).

Gr NP dispersions advantageously compared with Gr formulations in supported or non-supported cationic bilayers (Figure 7): the Gr/cationic bilayer formulations involved the hydrophobic effect between Gr and the cationic lipids in the bilayer, hampering to a certain extent Gr access to the pathogen. On the other hand, the weak interaction between Gr and PDDA hydrophilic polymer permitted facile access of Gr to the pathogen.

Previous reports on the incorporation of Gr as dimeric channels in cationic bilayers showed high activity against Gram-negative bacteria but did not yield the complete killing of Gram-positive ones or fungi [19,40]. Gr channels interacted strongly with the DODAB bilayer core via the hydrophobic effect [6,18], and the cationic bilayer was not able to completely kill Gram-positive bacteria or fungi. Here, the major novelty is the use of Gr combinations with PDDA against microbes in vitro. The interaction forces between the peptide NPs and the cationic polymer are much weaker than the ones between the peptide and the lipid bilayers, meaning a higher availability of both the peptide and the polymer to reach the microbes. In vivo, NPs injected in the circulation are rapidly opsonized and surrounded by the serum proteins, thereby remaining stable in the circulation until macrophages engulf them and they co-localize with the pathogenic microbes, killing them in locus [43]. The activity in vivo of the Gr NPs formulation described in this study will be evaluated in the future.

Hemolysis is a frequent problem when hydrophobic drugs, antibiotics, and antimicrobial peptides interact with red blood cells. In this respect, polymers, colloidal carriers, and hydrogels can introduce steric barriers to reduce the interaction between the agent and the cells [44]. PDDA alone does not cause hemolysis over a range of low concentrations ([PDDA] < 1 mg/mL) [22,45]. Although Gr is insoluble in water, here we show the occurrence of nanoparticles in water dispersion that were stable colloids and did not change their physical properties (Figure 3). Adding PDDA to the Gr NPs broadened the activity to also include Gram-negative bacteria (Table 2). Furthermore, minimal microbicidal concentrations (MMC) of each agent were reduced for Gr NPs/PDDA combinations (Table 2).

PDDA was previously shown to interact strongly with bacteria cell walls forming multilayers where PDDA and biopolymers of the cell wall were visualized as bundles protruding from the bacteria by scanning electron microscopy [33]. This might be the mechanism allowing Gr to more easily reach the cell membrane of bacteria and fungi: PDDA would weaken the bacteria and fungi cell walls, paving the way for Gr insertion in the microbial cell membranes. PDDA itself displayed remarkable microbicidal activity against Gram-negative bacteria and acted with Gr against Gram-positive ones and fungi, assuring not only the complete loss of viability against *S. aureus* and *C. albicans* but also reducing the doses of both agents against the fungus (Figure 9). In this respect, one must also refer to the use in vivo of PDDA, an excellent adjuvant to improve humoral response for vaccines; PDDA at 10 μg/mL yielded 90% of cell viability against mammalian macrophages and fibroblasts in culture [25]. Evidence from the literature has shown the penetration of cationic polymers into the microbial cell wall and membrane; the exposure of pathogenic bacteria to N-alkylated-poly ethylene imine coatings induced morphological damage and leakage of intracellular proteins [46,47] or phosphorylated molecules [33]; a hydrogel based on quaternized chitosan-graft-poly(ethylene glycol) methacrylate and poly(ethylene glycol) diacrylate displayed excellent antimicrobial efficacy against *Pseudomonas aeruginosa*, *Escherichia coli*, *Staphylococcus aureus*, and *Fusarium solani*, explained by the attraction of sections of anionic microbial membrane into the internal nanopores of the cationic hydrogel, leading to microbial membrane disruption and microbe death; the so-called sponge effect would suction out parts of the anionic cell wall into the gel [48]. Resistance mechanisms for antimicrobial peptides reported in the literature may involve the up-regulation of protease activity, reduction in pathogen negative surface charge, and release of extracellular anionic polysaccharides for scavenging of the cationic antimicrobials [49]. Agents that kill by disrupting or disturbing pathogen membranes, such as PDDA or Gr, do not target the microbe metabolic activity as often occurs with the development of resistance. Therefore, joining in a single formulation an antimicrobial peptide and an antimicrobial polymer yielded a promising combination worth testing also in vivo against animal models of infectious diseases.

A weak interaction of PDDA with Gr NPs was shown by the unchanged conductance of PDDA in the presence of the Gr NPs (Figure 6). As compared to the interaction between Gr and DODAB cationic bilayers driven by the hydrophobic effect with Gr insertion in the bilayer (Figure 7), the weak interaction between Gr and PDDA (Figure 6) allowed easy Gr and PDDA access to the microbes for activity at reduced doses (Figure 7). The DODAB bilayer reduced Gr activity because the hydrophobic bilayer microenvironment provided strong hydrophobic interactions between DODAB hydrocarbon chains and hydrophobic amino acid residues in the Gr molecule.

This light-scattering study describing the properties of Gr NPs can eventually become a model for studying other important aggregation events for proteins and peptides of clinical importance. In general, peptide self-assembly prevents their degradation through the protection of the cleavage sites and a reduction in the affinity for proteases [1]. Hydrophobic modification of peptides often drives the formation of supramolecular assemblies such as rod- or worm-like micelles or even nanoparticles as shown in this study for gramicidin (Figure 1 and Figure 2). Certain peptide amphiphile molecules self-assemble to yield a three-dimensional network of nanofibers that can act as an artificial but bioactive scaffold inducing the very rapid differentiation of cells into neurons thanks to the amplification of the bioactive epitope presentation to cells by the nanofibers [50]. The Gr NPs and Gr NPs/PDDA in the present study show good potential for further systemic uses due to their differential cytotoxicity (Table 2).

## 5. Conclusions

The supramolecular cationic assemblies of Gr NPs/PDDA exhibit a potent broad-spectrum microbicidal effect against Gram-negative and Gram-positive bacteria and fungus that takes place at reduced doses of both antimicrobials, and they do not affect mammalian cells such as macrophages or fibroblasts. Their mechanism of action depends on the facility of disassembly of PDDA from the Gr NPs; disassembly is important for the interaction between the pathogen and each agent so that the weak interactions between PDDA and Gr NPs favors the assembly of each agent with the pathogen. Once PDDA interacts with the outer cell wall of the pathogen, disassembling its biopolymers, the entrance of Gr into the pathogen cell membrane is facilitated. Using several formulations of cationic bilayers as carriers for Gr, a reduced Gr activity against the pathogens was determined due to the strong interaction between the hydrophobic residues of Gr and the lipid bilayers reducing Gr access to the pathogen. Therefore, the formulation of Gr NPs with PDDA, involving weak interactions between Gr NPs and PDDA, advantageously implemented a complementary activity of PDDA and Gr able to kill pathogens at remarkably reduced doses. The mechanical actions of PDDA suctioning the cell wall and of Gr entering the pathogen cell membrane to destroy the cell ionic balance establish a novel concept for formulating antimicrobial peptides and antimicrobial cationic polymers. The present study points out the need for further testing of the Gr NPs/PDDA formulation in vivo against models of infectious diseases.

## Figures and Tables

**Table 1:** Photos and physical properties of gramicidin (Gr) dispersions in water or in poly (diallyldimethyl ammonium chloride) (PDDA) water solutions. Physical properties were the mean z-average diameter (Dz), the zeta potential (ζ), the polydispersity (P), the conductance (G), and the turbidity at 400 nm. Gr dispersions were prepared from 0.031 mL of a 6.4 mM stock Gr solution in trifluoroethanol (TFE) added under stirring to 2 mL water. Gr/PDDA dispersions were prepared by adding 0.05 mL of a stock 10 mg/mL PDDA water solution to 2 mL of the Gr NP dispersion also under stirring. G of pure water was 5 ± 1 µS and G of TFE in water was 13 ± 1 µS.

Sample	Photo	Dz/nm	P	ζ/mV	G/µS	Turbidity/400 nm
0.25 mg/mL PDDA in water	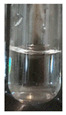	-	-	-	245 ± 10	0
0.1 mM Gr/TFE/water	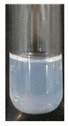	159 ± 1	0.14 ± 0.01	-26 ± 3	4 ± 2	0.43 ± 0.01
0.25 mg/mL PDDA/TFE/water	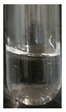	-	-	-	246 ± 11	0
0.1 mM Gr/0.25 mg/mL PDDA/TFE/water	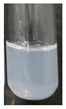	426 ± 31	0.24 ± 0.04	49 ± 1	236 ± 6	0.64 ± 0.01

**Table 2:** Differential cytotoxicity of Gr NPs, PDDA, and Gr NPs/PDDA combinations. The minimal microbicidal concentration (MMC) of each antimicrobial agent by itself or in dual combinations is given for Gr in µM and for PDDA in µg·mL^−1^. The reduction in cell viability is expressed as the number of logarithmic cycles (in between parentheses) besides each MMC. Asterisks refer to references * [25], ** [19], *** [22], and **** [33].

Cells	[Gr]/µM	[PDDA]/µg·mL^−1^	[Gr](µM)/[PDDA](µg·mL^−1^)
Macrophages/10^4^ cells		* 10 (0.05)	
Fibroblasts/10^4^ cells		* 10 (0.05)	
*E. coli*/10^8^ CFU	** 5 (0.3)	*** 5 (8)	
*P. aeruginosa*/10^7^ CFU		3 (7)	0.5/0.5 (7)
*P. aeruginosa* MDR/10^8^ CFU		**** 2 (8)	
*C. albicans/*10^7^ CFU	2 (7)	*** 5 (5)	0.5/0.5 (7)
*C. albicans* fluconazol R/10^5^ CFU		**** 1 (5)	
*S. aureus*	20 (7) (from 10^7^ to 1 CFU)	*** 10 (6) (from 10^8^ to 10^2^ CFU)	10/10 (6) (from 10^6^ to 1 CFU)

## Data Availability

All data available are reported in the article.
